# Predictive performance and metabolite dynamics of proton MR spectroscopy in neonatal hypoxic–ischemic encephalopathy

**DOI:** 10.1038/s41390-021-01626-z

**Published:** 2021-09-06

**Authors:** Hajnalka Barta, Agnes Jermendy, Livia Kovacs, Noemie Schiever, Gabor Rudas, Miklos Szabo

**Affiliations:** 1 Division of Neonatology, 1st Department of Paediatrics, Semmelweis University, Budapest, Hungary; 2 Medical Imaging Centre, Department of Neuroradiology, Semmelweis University, Budapest, Hungary

## Abstract

**Background:**

Prognostic value of proton MR spectroscopy (H-MRS) in hypoxic–ischemic encephalopathy (HIE) is acknowledged; however, effects of gestational age (GA) and postnatal age (PA) on prediction and metabolite levels are unknown.

**Methods:**

One hundred and sixty-nine newborns with moderate-to-severe HIE were studied, having ≥1 H-MRS scan during postnatal days 0–14 and known neurodevelopmental outcome (Bayley-II score/cerebral palsy/death). Initial scans were categorized by PA (day 1–3/4–6/≥7), and metabolite ratios were compared by predictive value. Metabolite dynamics were assessed in a total of 214 scans performed in the study population, using regression modeling, with predictors GA, PA, and outcome.

**Results:**

*N*-acetyl-aspartate (NAA)/creatine (Cr) and myo-inositol (mI)/NAA height ratios were consistently associated with outcome throughout the first 14 days, with the highest predictive value in the late (≥7 days) period (AUC = 0.963 and 0.816, respectively). Neither GA nor PA had an overall effect on these metabolite ratios, which showed strongest association with outcome (*p* < 0.001). Assessed separately in patients with good outcome, GA became a significant covariate for metabolite ratios (*p* = 0.0058 and 0.0002, respectively). However, this association disappeared in the poor outcome group.

**Conclusions:**

In HIE, NAA/Cr and mI/NAA give most accurate outcome prediction throughout postnatal days 0–14. GA only affected metabolite levels in the good outcome group.

**Impact:**

- Proton MR spectroscopy metabolite ratios *N*-acetyl-aspartate/creatine and myo-inositol/*N*-acetyl-aspartate have persistently high predictive value throughout postnatal days 0–14 in newborns with hypoxic–ischemic encephalopathy, with the highest predictive power between postnatal days 7 and 14.
- Overall, neither metabolite ratio was affected by gestational age nor by postnatal age, while they showed the strongest association with neurological outcome.
- However, in newborns facing good outcome, metabolite ratios were associated with gestational age, whereas in cases facing poor outcome, this association disappeared.
- Proton MR spectroscopy provides valuable prognostic information in neonatal hypoxic–ischemic encephalopathy throughout the first 2 weeks of life, irrespective of the timing of MR scan.

## Introduction

Neonatal hypoxic–ischemic encephalopathy (HIE) affects 1–2‰ of newborns in the developed countries and is one of the leading causes of permanent neurological handicap^1^. A wide variety of neuroprotective therapies are emerging, additional to the current gold standard therapeutic hypothermia, emphasizing the need for early outcome prediction for risk stratification strategies^2,3^.

Brain proton magnetic resonance spectroscopy (H-MRS) is one of the most accurate tools for predicting long-term neurodevelopmental outcome in HIE, especially during the first weeks of life^4,5^. Recent evidence suggests that the prognostic accuracy of H-MRS is not equal in the period between postnatal hours 18–96 and postnatal days 7–14^6^. However, the effect of the newborns’ postnatal age on the predictive value of H-MRS throughout the first weeks of life has not been studied in detail, albeit examination of all newborns with HIE in a uniform postnatal narrow age window is not plausible, due to variable clinical stability and available resources. Moreover, existing evidence proposes that metabolite ratios may also be affected by gestational age^7^. The influence of neither of these factors has been studied so far on the predictive value of H-MRS, even though accurate description of metabolite dynamics would be essential, especially by investigating metabolite ratios registered conventionally, to ensure generalizability.

Thus, the purpose of our study was to determine whether H-MRS is a suitable predictive tool throughout days of life 0–14 with particular interest in the effect of postnatal age at H-MRS scan on the accuracy of outcome prediction in newborns with HIE. Additionally, we aimed to investigate how gestational age and postnatal age affect the most commonly analyzed, conventionally determined metabolite ratios.

## Methods

### Patient selection

In our retrospective analysis, we reviewed all 484 consecutive patients diagnosed with moderate-to-severe HIE according to the criteria of the international TOBY trial^8^, born between January 2006 and December 2016, and admitted to the regional cooling center, the Neonatal Intensive Care Unit (NICU) of the 1st Department of Paediatrics, Semmelweis University, Budapest, Hungary. Prospective consent was obtained from parents/legal guardians of all newborns participating in our study. Ethical permission for the analysis was obtained from the Scientific and Medical Research Council Ethics Committee of Hungary (11790-2/2016/EKU).

**Fig. 1:**
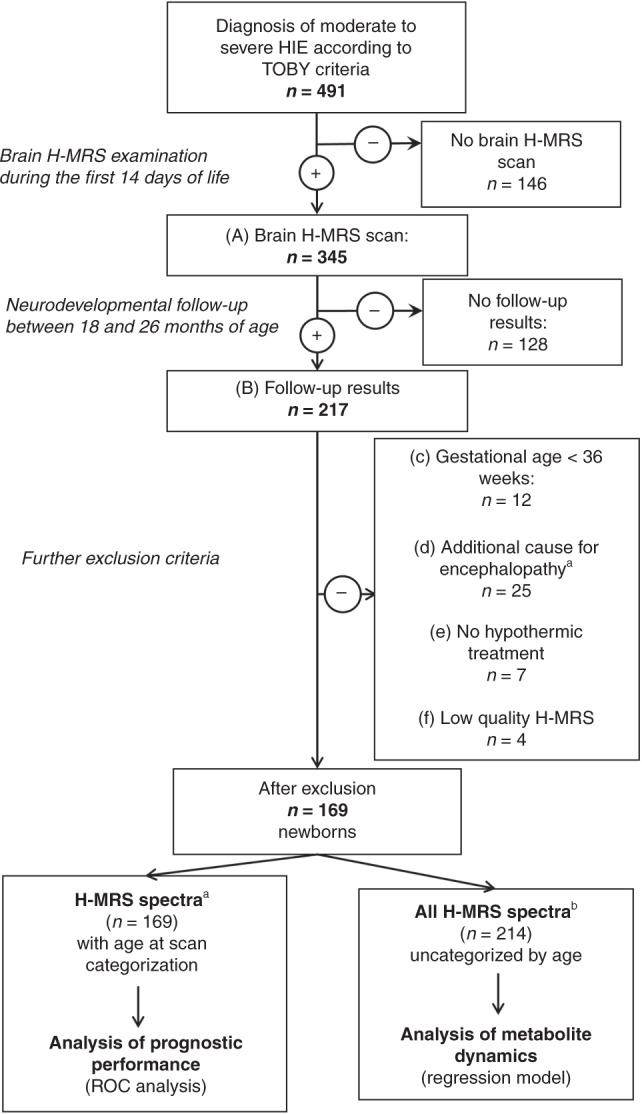
Algorithm of patient selection for our study, detailing inclusion and exclusion criteria. ^a^Age at scan categorization was done based on the initial performed H-MRS scan, where more than one was performed. ^b^Initial and control H-MRS spectra were also included, without age at scan categorization. ROC receiver-operating characteristics.

Inclusion criteria were (A) having at least one brain H-MRS examination performed during the first 14 days of life and (B) having a neurodevelopmental follow-up examination result, as detailed below OR death (<28 days of life OR >28 days associated with HIE). Exclusion criteria were (c) gestational age <36 weeks OR (d) other underlying conditions for encephalopathy besides HIE (i.e., stroke, intracranial hemorrhage, congenital malformation, or metabolic disease) OR (e) lack of hypothermia treatment OR (f) low-quality brain H-MRS scan. The algorithm for patient selection is presented in Fig. 1.

### Clinical care of newborns

Whole-body hypothermia treatment was initiated as soon as possible but within 6 h after delivery using a water-filled mattress (Tecotherm©; TecCom, Halle, Germany). The target rectal temperature was between 33 and 34 °C, maintained for 72 h. In the rewarming phase, temperature increase velocity was 0.5 °C/h. All infants were invasively ventilated throughout the cooling and rewarming phase and were extubated following rewarming. Sedation was provided using continuous morphine infusion, and clinical or electrophysiological seizures were treated with phenobarbitone and midazolam, if necessary.

Clinical care of the newborns was performed according to our unit’s protocol for HIE in the examined time period, described in detail in our previous study^9^. Neurological staging was performed according to the modified Sarnat amplitude-integrated electroencephalography (aEEG) scoring system, as follows: moderate encephalopathy was defined as moderately abnormal aEEG pattern (discontinuous normal voltage) on <6 h aEEG AND Sarnat stage 1–2, severe encephalopathy was defined as severely abnormal (burst suppression (BS), low voltage (LV), or flat trace (FT)) on <6 h aEEG OR Sarnat 3^10^. In case of hemodynamic instability, infants received cardiovascular support (dobutamine, dopamine, norepinephrine, epinephrine, hydrocortisone, or milrinone) and invasive ventilation.

### H-MRS examination

H-MRS studies were carried out as part of the routine MR examination on a 3-Tesla Philips Achieva magnetic resonance imaging (MRI) scanner between 2006 January and 2015 November and on a 3-Tesla Philips Ingenia MRI scanner between 2015 December and 2016 December (Philips Medical Systems, Best, The Netherlands) at the Department of Neuroradiology of the Medical Imaging Centre, Semmelweis University, as early as the infant reached clinical stability and was suitable for transport. Control MR and H-MRS were performed at a later age, if needed. The MR scans were performed between days of life (DOL) 1 and 14 (median 4).

The Neonatal Emergency & Transport Services of the Peter Cerny Foundation provided the neonatal transport and the critical care, including hypothermia treatment and sedation during MR scanning. Continuous monitoring of transcutaneous oxygen saturation and capnography was provided for all neonates during the MR scan, using Medrad Veris MR Monitoring System (Bayer Healthcare LLC, Whippany, NJ). During the MR examination, passive hypothermia was provided (bare infants, covered only with a blanket), considering the relatively cool room temperature of the MR examination room. Pre-scan and post-scan core temperature was checked per protocol, and no deviation larger than ±0.5 °C was detected.

### Acquisition protocols

**Fig. 2:**
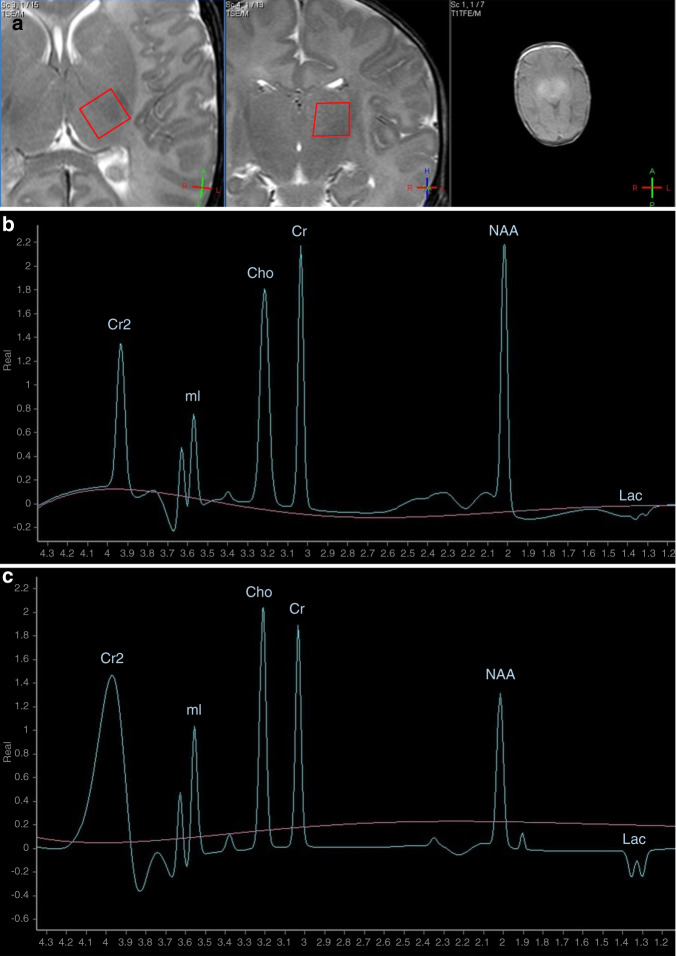
Representative H-MRS spectra of newborns with moderate-to-severe HIE. **a** Gradient echo survey images acquired with echo time (TE) = 5 ms, repetition time (TR) = 75 ms, and 30° flip angle for selection of volume of interest (VOI) from the left thalamus of infants (red frame). **b** H-MRS of newborn who later had good outcome at TE = 144 ms. **c** H-MRS of newborn who later had poor outcome at TE = 144 ms. Cho choline, Cr creatine, Cr2 creatine, second peak, Lac lactate, doublet peak, mI myo-inositol, doublet peak, NAA *N*-acetyl-aspartate. *x* axis represents chemical shift in parts per million (ppm), *y* axis represents signal intensity in arbitrary units.

Proton MR spectra were acquired using the PRESS (Point RESolved Spectroscopy) single-voxel localization sequence, at an intermediate echo time (TE) of 144 ms, repetition time (TR) = 2000, number of signal acquisitions (NSA) = 128. Duration of scan was approximately 30 min. The analyzed volume of interest was a 1 × 1 × 1 cm voxel in the left thalamus of infants, localized based on gradient echo survey images acquired with TE = 5 ms, TR = 75 ms, and 30° flip angle (Fig. 2a). For analysis of the acquired spectra, we used the vendor-provided data-processing software on the MR console, “Spektroview,” and its generic scripts, consisting of the following data-processing steps: (1) Initial Baseline Subtraction (only on 144 and 288 ms TEs), (2) Peak Selection (where all possible peaks were selected, except the water peak), (3) Peak Fitting (Analysis range [−1; 4.35] ppm, Visualized range [0; 4.35] ppm, Baseline Terms: 7; Gaussian Character [%]: 90), (4) Phase: Real, (5) Lock relative frequency option: ON (this enables for more robust peak fitting, by detecting the individual peaks using their known difference of frequency), (6) Lock width option: OFF (peak width is freely fitted by the software).

Representative spectra of newborns facing good and poor outcome are presented in Fig. 2b, c.

### Metabolite ratios

For quality assurance of the registered H-MRS, only spectral data of peaks with sufficiently high signal-to-noise ratio (>2) were included in our analysis. From the registered H-MRS spectrum, we analyzed the metabolite ratios showing high predictive power in newborns with HIE when registered conventionally, according to existing research results: *N*-acetyl-aspartate (NAA)/creatine (Cr), NAA/choline (Cho), and myo-inositol (mI)/NAA ratios^9^. Both ratios of peak height and ratios of peak areas were analyzed, due to their similar reliability in case of conventional H-MRS sequence^11^.

### Postnatal age categories

For analysis of optimal postnatal age at H-MRS for outcome prediction, first acquired H-MRS results were categorized based on postnatal age at scan, given in DOL, as follows: (I) early H-MRS (DOL 1–3), (II) midtime H-MRS (DOL 4–6) and (III) late H-MRS (DOL ≥7)^12^.

In the analysis of metabolite ratio dynamics, initial and control H-MRS results were also included, without the above-described age categorization.

### Follow-up

Neurodevelopmental follow-up was measured by Bayley Scales of Infant Development II tool-kit (BSID-II) on all patients performed between 18 and 26 months of age by trained personnel blinded to the H-MRS results. We defined poor outcome as a composite of either death (<28 days of age OR >28 days associated with HIE) OR moderately/severely delayed development (Mental Developmental Index (MDI) or Psychomotor Developmental Index (PDI) <70). In children where BSID-II was not feasible due to severe disability, diagnosed cerebral palsy OR bilateral cortical visual impairment with no useful vision OR bilateral sensorineural hearing loss constituted the poor outcome. All other outcomes were considered as good outcome.

### Statistical analysis

Categorical variables are reported as absolute numbers and percentages, while continuous variables as mean ± standard deviation or median [25th to 75th interquartile range] depending on the distribution of the parameters. Shapiro–Wilk test was used to assess normality. Categorical variables were compared with Fisher’s exact test, while continuous variables were compared with Student’s *t* test/analysis of variance or Mann–Whitney *U* test/Kruskal–Wallis test for parametric and non-parametric comparisons, respectively. Prognostic performance of the metabolite ratios showing significant association with outcome was evaluated by receiver-operating characteristics (ROC) analysis. Repeated-measures linear mixed-effect regression model, with first-order autoregressive within-group correlation structure fitted by maximizing the restricted log-likelihood, was performed to assess metabolite dynamics, with metabolite ratios as dependent variables and postnatal age, gestational age, and neurological outcome as predictors. Demographic, clinical, and spectral data were analyzed using the IBM SPSS Statistics software version 23.0.0.0 (IBM Corporation, Armonk, NY), as well as R Statistical software 4.0.2 (R Core Team, Vienna, Austria).

## Results

One hundred and sixty-nine neonates with moderate-to-severe HIE met the inclusion criteria, and were enrolled in our study. Patient selection is presented on Fig. 1. From this patient pool, 129 newborns had only 1 initial H-MRS scan, 30 newborn had a total of 2 scans, and 8 newborn had 3 scans, summing to a total of 214 H-MRS results. Initial scans were performed on median DOL 4, second scans on median DOL 7, and the third scans on median DOL 8. Exact metabolite ratios for newborns having multiple H-MRS scans are presented in the Supplementary Table.

A total of 7 newborns were excluded due to lack of hypothermia treatment as per protocol: 1 infant did not receive cooling as it was not yet adopted as a standard center practice in 2008, 1 infant received hypothermia with late onset (9 h of life) due to logistical reasons, and 5 newborns suffered postpartum asphyxia treated with altered hypothermia protocols. All other 169 infants enrolled received therapeutic hypothermia per protocol.

Ninety-six of the 169 patients had the H-MRS examination performed while receiving whole-body hypothermia; the remaining 73 patients were scanned under normothermic conditions, after the completion of hypothermia treatment. All newborns with early (DOL 1–3) H-MRS scans had the examination under hypothermic conditions, and 6 newborns with midtime (DOL 4–6) H-MRS scan were examined during ongoing hypothermic treatment.

A total of 69 patients had poor outcome, among whom 25 died, all during the neonatal period. Of the 44 newborns who survived but presented with poor outcome, MDI <70 was registered in 40 cases, whereas PDI <70 in 37 cases (both scores where <70 in 35 cases). Cerebral palsy was diagnosed in 21 cases, and 1 child presented with bilateral sensorineural hearing loss.

Clinical data of newborns are presented in Table 1 according to postnatal age category at initial H-MRS, as well as in Table 2, sorted according to the number of performed H-MRS examinations.

**Table 1:** Clinical characteristics of newborns enrolled in the study, categorized by postnatal age at initial scan.

	Early H-MRS (DOL 1–3), *n* = 91	Midtime H-MRS (DOL 4–6), *n* = 50	Late H-MRS (DOL 7–14), *n* = 28	*p* value
Male sex, %	63%	37%	61%	0.071
Gestational age (weeks)	39 [38; 40]	40 [39; 40]	39 [37; 40]	0.298
Birth weight (g)	3175 ± 521	3321 ± 542	3082 ± 528	0.126
Apgar 1’	2 [0; 3]	2 [1; 4]	2 [1; 5]	0.425
Apgar 5’	4 [3; 6]	5 [3; 6]	5 [3; 7]	0.107
Apgar 10’	5 [4; 7]	6 [5; 7]	5 [4; 7]	0.758
Onset of hypothermia (h)	1.8 [1.2; 2.5]	2.1 [1.4; 2.6]	1.8 [1.0; 2.7]	0.439
Lowest pH <1 h of age	7.03 ± 0.20	7.04 ± 0.18	6.96 ± 0.24	0.202
Highest BD <1 h of age	17.7 ± 6.2	16.1 ± 6.7	16.5 ± 7.0	0.352
Neurology				
Clinical or aEEG seizures (<24 h)	59%	56%	39%	0.173
Abnormal aEEG pattern (DNV, BS, LV, FT)	98%	94%	96%	0.506
aEEG normalization (CNV) <72 h	38%	58%	43%	0.805
aEEG normalization time (h)	20 [7; 44]	14 [10; 32]	10 [5;41]	0.533
Severity of encephalopathy (severe)^a^	85%	68%	71%	0.054
Cardiorespiratory status and multiorgan failure				
Need for invasive ventilation^b^	93%	94%	93%	0.980
Length of invasive ventilation (days)	4 [4; 5]	5 [4; 5]	5 [5; 7]	0.018*
Need for cardiovascular support^c^	65%	74%	82%	0.172
Length of cardiovascular support (h)^d^	67 [40; 146]	135 [74; 197.5]	163 [60; 229]	0.002*

Data shown as median [IQR], mean ± SD, or percentage.

*BD* base deficit, *aEEG* amplitude-integrated electroencephalography, *BS* burst suppression, *LV* low voltage, *FT* flat trace, *CNV* continuous normal voltage, *DNV* discontinuous normal voltage.

*Bold figures represent significant results of chi-square test, ANOVA, or Kruskal–Wallis test (*p* < 0.05).

^a^Moderate and severe encephalopathy defined using the modified Sarnat aEEG score^10^, as follows: moderate encephalopathy DNV on <6 h aEEG AND Sarnat stage 1–2, severe encephalopathy: BS, LV, or FT on <6 h aEEG OR Sarnat 3.

^b^SIMV (synchronized intermittent mandatory ventilation), SIPPV (synchronous positive pressure ventilation) or HFO (high frequency oscillatory ventilation).

^c^Dobutamine, dopamine, norepinephrine, epinephrine or milrinone infusion, or hydrocortisone supplementation.

^d^Calculated by summing up the length for the different vasoactive medications, in hours.

**Table 2:** Clinical characteristics of newborns, categorized by postnatal age at initial scan.

	Single H-MRS performed, *n* = 135	Multiple (2 or 3) H-MRS performed, *n* = 34	*p* value
Male sex, %	58%	62%	0.702
Gestational age (weeks)	40 [38; 40]	39 [38; 40]	0.535
Birth weight (g)	3230 ± 510	3090 ± 600	0.179
Apgar 1’	1 [0; 4]	2 [1; 4]	0.100
Apgar 5’	5 [3; 6]	4 [3; 6]	0.998
Apgar 10’	5 [4; 7]	5 [4; 7]	0.915
Onset of hypothermia (h)	1.8 [1.2; 2.6]	1.8 [1.2; 2.8]	0.673
Lowest pH <1 h of age	7.01 ± 0.21	7.07 ± 0.17	0.135
Highest BD <1 h of age	17.0 ± 6.6	17.1 ± 6.1	0.946
Neurology			
Clinical or aEEG seizures (<24 h)	67%	85%	0.527
Abnormal aEEG pattern (DNV, BS, LV, FT)	95%	100%	0.601
aEEG normalization (CNV) <72 h	72%	72%	>0.999
aEEG normalization time (h)	14 [7; 36]	36 [12; 44]	0.250
Severity of encephalopathy (severe)^a^	75%	88%	0.111
Cardiorespiratory status and multiorgan failure			
Need for invasive ventilation^b^	93%	97%	0.696
Length of invasive ventilation (days)	5 [4; 5]	4 [4; 5]	0.601
Need for cardiovascular support^c^	70%	71%	>0.999
Length of cardiovascular support (h)^d^	116 [60; 206]	64 [31; 131]	0.004*

Data shown as median [IQR], mean ± SD, or percentage.

*BD* base deficit, *aEEG* amplitude-integrated electroencephalography, *DNV* discontinuous normal voltage, *BS* burst suppression, *LV* low voltage, *FT* flat trace, *CNV* continuous normal voltage, *DNV* discontinuous normal voltage.

*Bold figures represent significant results of chi-square test, ANOVA, or Kruskal–Wallis test (*p* < 0.05).

^a^Moderate and severe encephalopathy defined using the modified Sarnat aEEG score^10^, as follows: moderate encephalopathy DNV on <6 h aEEG AND Sarnat stage 1–2, severe encephalopathy: BS, LV, or FT on <6 h aEEG OR Sarnat 3.

^b^SIMV (synchronized intermittent mandatory ventilation), SIPPV (synchronous positive pressure ventilation) or HFO (high frequency oscillatory ventilation).

^c^Dobutamine, dopamine, norepinephrine, epinephrine or milrinone infusion, or hydrocortisone supplementation.

^d^Calculated by summing up the length for the different vasoactive medications, in hours.

**Fig. 3:**
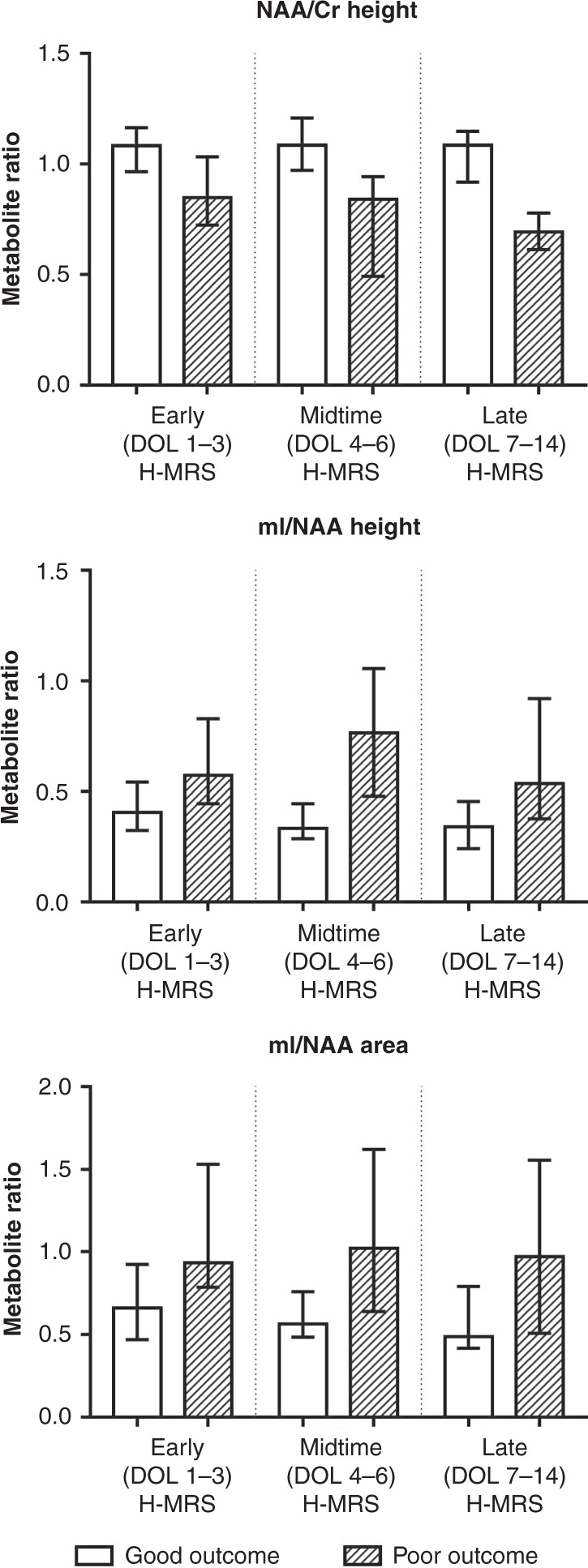
H-MRS metabolite ratios showing association with outcome across all postnatal age categories (postnatal days 0–14), categorized by outcome and postnatal age category. Bars represent medians, error bars represent interquartile ranges. mI myo-inositol, NAA *N*-acetyl aspartate, DOL day of life.

Analysis of the association between metabolite ratios and outcome across all three postnatal age categories revealed that only NAA/Cr peak height ratio and mI/NAA peak height ratio and peak area ratio were able to consistently differentiate between outcomes throughout early, midtime, and late postnatal age windows as well. Detailed results of the performed Mann–Whitney *U* test are presented in Table 3 and Fig. 3. ROC analysis showed that the highest predictive value could be observed in the late (DOL ≥ 7) postnatal age window for both NAA/Cr peak height and mI/NAA peak height and peak area ratios. Overall predictive value was higher for mI/NAA height ratio than for mI/NAA area ratio (calculated as a sum of areas under the ROC curve across postnatal age categories), therefore we aimed the mixed-effect regression model on NAA/Cr height ratio and mI/NAA height ratio. Detailed results of the performed Mann–Whitney *U* test and ROC analysis are displayed in Table 4.

**Table 3:** Results of Mann–Whitney *U* test comparison of metabolite ratios and outcome, across postnatal age categories at H-MRS scan (*n* = 169).

	Early H-MRS (DOL 1–3)			Midtime H-MRS (DOL 4–6)			Late H-MRS (DOL ≥ 7)		
	Good *n* = 44	Poor *n* = 47	*p* value	Good *n* = 37	Poor *n* = 13	*p* value	Good *n* = 19	Poor n = 9	*p* value
NAA/Cr height	1.08 [0.97; 1.16]	0.85 [0.73; 1.02]	<0.0005*	1.09 [0.99; 1.21]	0.84 [0.52; 0.94]	<0.0005*	1.09 [0.93; 1.15]	0.69 [0.62; 0.74]	<0.0005*
NAA/Cr area	1.05 [0.94; 1.15]	0.91 [0.78; 1.02]	<0.0005*	1.00 [0.97; 1.12]	0.93 [0.67; 1.08]	0.111	0.98 [0.83; 1.12]	0.72 [0.66; 0.76]	<0.0005*
NAA/Cho height	0.95 [0.88; 1.09]	0.86 [0.74; 1.03]	0.097	0.80 [0.75; 0.92]	0.69 [0.57; 0.83]	0.167	0.65 [0.56; 0.72]	0.48 [0.42; 0.66]	0.053
NAA/Cho area	0.69 [0.62; 0.77]	0.65 [0.53; 0.75]	0.088	0.70 [0.66; 0.73]	0.55 [0.40; 0.59]	<0.0005*	0.58 [0.49; 0.65]	0.38 [0.37; 0.45]	0.001*
mI/NAA height	0.41 [0.33; 0.52]	0.57 [0.45; 0.81]	<0.0005*	0.33 [0.29; 0.44]	0.77 [0.50; 1.01]	<0.0005*	0.34 [0.24; 0.44]	0.54 [0.41; 0.89]	0.009*
mI/NAA area	0.66 [0.48; 0.91]	0.94 [0.80; 1.51]	0.004*	0.56 [0.49; 0.74]	1.02 [0.71; 1.51]	0.002*	0.49 [0.42; 0.79]	0.97 [0.53; 1.37]	0.009*

Data shown as median [IQR].

*DOL* day of life, *NAA* *N*-acetyl-aspartate, *Cr* creatine, *Cho* choline, *mI* myo-inositol.

*Bold figures represent significant results (*p* < 0.05).

**Table 4:** Results of Mann–Whitney *U* test comparison of metabolite ratios and outcome, across postnatal age categories at H-MRS scan, as well as receiver-operating characteristics (ROC) analysis of metabolite ratios.

	Early H-MRS (DOL 1–3)				Midtime H-MRS (DOL 4–6)				Late H-MRS (DOL ≥7)			
	AUC	Cutoff	SENS	SPEC	AUC	Cutoff	SENS	SPEC	AUC	Cutoff	SENS	SPEC
NAA/Cr height	0.807	0.96	81.8%	70.2%	0.871	0.95	81.1%	84.6%	0.963	0.83	88.9%	88.9%
NAA/Cr area	0.733	0.99	70.5%	68.1%	0.65	0.69	97.3%	46.2%	0.895	0.78	94.4%	77.8%
NAA/Cho height	0.601	0.85	77.3%	48.9%	0.63	0.69	86.5%	53.8%	0.735	0.54	88.9%	66.7%
NAA/Cr area	0.604	0.59	84.1%	42.6%	0.84	0.65	78.4%	84.6%	0.883	0.45	94.4%	77.8%
mI/NAA height	0.719	0.42	59.0%	82.6%	0.897	0.37	63.6%	99.9%	0.816	0.42	73.7%	75.0%
mI/NAA area	0.683	0.80	69.2%	72.7%	0.818	0.85	84.8%	70.0%	0.816	0.93	99.9%	62.5%

*AUC* area under ROC curve, *SENS* sensitivity, *SPEC* specificity.

A mixed-effect regression model was constructed to investigate metabolite dynamics in the whole patient population, using NAA/Cr and mI/NAA height ratios as dependent variables and gestational age, postnatal age and neurological outcome as predictors. Ongoing hypothermia treatment was not included as a covariate, as it is coincidental with postnatal age during the first 3 postnatal days, due to the therapeutic hypothermia protocol (onset of hypothermia <6 postnatal hours, duration of hypothermia 72 h)^8^. We found that, overall in the whole study population, outcome was the only significant covariate for both metabolite ratios, suggesting that these metabolite ratios are not affected by gestational age and postnatal age.

However, analyzing the mixed-effect model separately in the two outcome groups revealed that both NAA/Cr and mI/NAA ratios are significantly affected by gestational age in the good outcome group, but this association disappears in the poor outcome group. In case of postnatal age though, in the good outcome group, mI/NAA metabolite ratio proved to be highly associated with postnatal age but not NAA/Cr. In the poor outcome group, NAA/Cr showed only slight association with postnatal age, whereas mI/NAA none. Results of the regression analysis are presented in Table 5.

**Table 5:** Effect of gestational age, postnatal age, and neurological outcome on metabolite dynamics during the first 14 days of life.

	NAA/Cr height			mI/NAA height		
	Coefficient	95% CI	*p* value	Coefficient	95% CI	*p* value
General						
(Intercept)	0.4522	(−0.3335, 1.2379)	0.2574	1.1115	(−0.2324, 2.4553)	0.1043
Gestational age (weeks)	0.0162	(−0.0038, 0.0362)	0.1115	−0.0177	(−0.0519, 0.0165)	0.3086
Postnatal age (days)	−0.0450	(−0.1015, 0.0114)	0.1148	−0.0467	(−0.1224, 0.0291)	0.2209
Poor outcome	−0.2277	(−0.2883, −0.1672)	0.0000*	0.3167	(0.2125, 0.4208)	0.0000*
Good outcome group						
(Intercept)	−0.1929	(−1.0761, 0.6902)	0.6656	1.8131	(1.1285, 2.4978)	0.0000
Gestational age (weeks)	0.0320	(0.0095, 0.0545)	0.0058*	−0.0346	(−0.0521, −0.0171)	0.0002*
Postnatal age (days)	−0.0038	(−0.0723, 0.0646)	0.9088	−0.1047	(−0.1601, −0.0494)	0.0007*
Poor outcome group						
(Intercept)	0.7541	(−0.5712, 2.0795)	0.2600	0.5655	(−2.3818, 3.5128)	0.7025
Gestational age (weeks)	0.0032	(−0.0310, 0.0374)	0.8513	0.0039	(−0.0723, 0.0802)	0.9182
Postnatal age (days)	−0.0958	(−0.1824, −0.0091)	0.0319*	0.0151	(−0.1133, 0.1435)	0.8083

Results of mixed-effect regression models are shown, presented overall in the whole study population and separately in the good and poor outcome groups.

*95% CI* confidence interval (at 95% confidence level).

*Bold figures represent significant results (*p* < 0.05).

## Discussion

To the best of our knowledge, this is the first study to examine the effect of postnatal age on the predictive value of brain H-MRS in newborns with HIE throughout the first 2 weeks of life, as well as the combined effect of gestational age, postnatal age, and outcome on metabolite ratios. Our findings suggest that H-MRS is a valuable predictive tool throughout the examined postnatal age windows—the first 14 days of life—with the highest predictive power between postnatal days 7–14, for the metabolite ratios showing the strongest prognostic power, namely, NAA/Cr and mI/NAA ratios. Moreover, these two metabolite ratios showed the strongest association with neurodevelopmental outcome and were not significantly affected by gestational age and postnatal age, overall.

In our previous study, assessment of early (first 96 h of life), conventional H-MRS concluded that, other than having predictive power, NAA/Cr, NAA/Cho, and mI/NAA show weak correlation with postnatal age at scan during first 4 days of life, hence might be considered relatively stable in this early postnatal age period^9^. However, no examination has been performed concerning the postnatal age dependence of these metabolite ratios in the later postnatal period. In the current research, we hypothesized that after being relatively stable in the first 4 days of life, dynamic changes of these metabolite ratios do appear in the later time periods, potentially modifying their predictive power. Our results suggest that, whereas prognostic accuracy of these metabolite ratios varies with age, NAA/Cr and mI/NAA keep their predictive power throughout the first 14 days of life, making them optimal for prediction.

The physiological and gestational age-related variability of metabolite levels in healthy fetuses has been previously described in detail. According to Urbanik et al., a significant rise can be detected in NAA, Cr, Cho, and mI absolute concentrations throughout gestation (weeks 18–40)^7^. This observation is partly confirmed by Kok et al., also describing increasing NAA absolute levels between gestational weeks 30–41, further adding that, while NAA/Cr and NAA/Cho ratios also rise in the examined gestational period, Cho/Cr ratio decreases, suggesting that absolute metabolite levels do not elevate uniformly and with the same velocity throughout physiological neuronal maturation^13^.

The results of our mixed-effect regression model propose that, when outcome is taken into consideration as a covariate, neither gestational age nor postnatal age have a significant effect on NAA/Cr and mI/NAA metabolite ratios. This suggests that the moderate-to-severe hypoxic–ischemic injury has such a definitive impact on brain metabolism (and consequently, metabolite levels and ratios) that the influence of other modifying factors like gestational age or postnatal age disappears.

However, when these associations were analyzed in the good outcome group alone, it was revealed that these metabolite ratios are greatly influenced by gestational age—similar to healthy newborn conditions—represented by a rise in NAA/Cr ratio and a decrease in mI/NAA ratio, as gestational age increases. Additionally, in the good outcome group, postnatal age turned out to be a significant covariate, but only for mI/NAA, represented by a decrease in mI/NAA with increasing postnatal age. This might imply that, in case of newborns facing good outcome, the physiological, gestational age-dependent variability of metabolite levels can be observed, despite the HIE (presumably due to having undergone milder neuronal injury). Moreover, in case of mI/NAA, signs of postnatal age-dependent metabolic variability also emerged.

Based on our observation, in the poor outcome group though, the effect of gestational age on metabolite ratios disappeared, suggesting that the more severe metabolic changes caused by a more intense hypoxic–ischemic insult may obscure the physiological pattern evolution of metabolites. Furthermore, the effect of postnatal age on mI/NAA ratio also vanished, whereas NAA/Cr ratio presented an although significant but slight association with postnatal age.

A number of existing research papers studying H-MRS as a prognostic tool in HIE use various methods for data optimization (e.g., custom made headcoil, absolute quantification sequences, post-processing techniques)^14–16^. These methods enable the accurate detection and measurement of the lactate (Lac) metabolite peak, making Lac and Lac-derived ratios (Lac/NAA, Lac/Cr, Lac/Cho) also among the most widely analyzed metabolites for prediction. While the above listed methods may indeed improve data quality, these methods may not be generally applicable in all clinical settings, especially in those with low resources. Our aim was to assess whether H-MRS is an adequate prognostic tool, as well as to describe the dynamics of conventional H-MRS, throughout the first 14 days of newborns with HIE, for our conclusions to be applicable in every clinical setting. Nevertheless, based on our previous study, Lac peak cannot be accurately detected under conventional conditions, due to high noise levels^9^. Additionally, it has been previously described that at 3 T and moderate TE (e.g., 144 ms), Lac peak suffers signal loss and may not be detectable due to anomalous j-coupling^17^. Also, Wu et al. found that in newborns with moderate and severe HIE, cerebral Lac concentrations peak early in all examined cerebral regions (basal ganglia, thalamus, cortex, and white matter) and, in most cases, fall in the normal range after postnatal days 4–5^18^. Moreover, recent evidence suggests that Lac ceases to be an accurate H-MRS predictor, when analyzed in newborns treated with therapeutic hypothermia, an observation presumably indicating the working mechanism of hypothermic treatment for HIE^19,20^. Conclusively, for the sake of our initial aim, we excluded Lac from the list of our metabolite ratio candidates.

While NAA and NAA/Cr ratio are extensively studied biomarkers, few studies focus on mI and its significance in HIE^21^. mI, as a conformation isomer of the pentose sugar inositol, is one of the main osmolites of human cells, as well as an intracellular signal transmitter and precursor molecule for lipid synthesis. During gestation and maturation, its level rises around term (with ongoing myelination), being one of the highest peaks in the neonatal period, and then rapidly decreases with age, reaching its final level around 1 year of age. Being a membrane compound, its level significantly rises after neuronal injuries, suggesting cell death^22,23^.

Current guidelines for management of HIE recommend the performance of more than one MR examination^12^, considering that the utility and sensitivity of its various imaging modalities change with the postnatal age of the newborns—conclusively, H-MRS of asphyxiated newborns is also performed at various postnatal ages. Moreover, H-MRS examination of all newborns with HIE in a uniform postnatal narrow age window is not possible, due to the variable clinical stability of neonates with HIE, as well as resource availability. This further emphasizes the importance of detailed description of how postnatal age affects metabolite ratios and, more importantly, their predictive power. According to our study, it seems that H-MRS examination keeps its outstanding predictive value throughout the first 14 days of life in newborns with HIE. Therefore, performing an additional H-MRS sequence whenever a brain MRI examination is done provides valuable additional diagnostic and prognostic information to the clinician.

### Limitations

Despite our careful preparation of our study methodology, there are limitations that should be taken into consideration. The general limitation of our study is the lack of healthy, gestational age-specific reference data from the first 2 weeks of the neonatal period. However, this would be an unrealistic expectation, from both ethical and methodological reasons, and theoretically, this missing referential data is not indispensable for prognostication. The other limitation is the retrospective nature of the study. On the one hand, this is a limitation compared to a prospective study with fine-tuned imaging and clinical parameters for research purposes; however, on the other hand, this could be viewed as a strength from a clinical point of view, having relied on data obtainable from any MR facility dealing with asphyxiated neonates, thus being more generalizable. Furthermore, due to resource availability, two different 3 Tesla MRI scanners were used to examine our patient population. While this might bias the acquired spectral data, this technical issue is counteracted by the identical magnetic field strength of the scanners, as well as our assessment of metabolite ratios instead of absolute metabolite levels. Our results could also be biased by the uneven sample sizes of our patient groups, which may decrease the accuracy of our statistical analysis. Another potential limitation might be the fact that a large number of patients were unavailable to follow-up and had to be excluded from the outcome analysis. However, clinical parameters of surviving newborns lost to follow-up did not differ significantly from those with follow-up results (data not shown), therefore a systematic bias is unlikely. Moreover, the establishment of postnatal age categories by itself, for analysis of prognostic performance of HMRS, can be considered a limitation, since it inevitably creates artificial conditions. While assessment of postnatal age at scan in considerably smaller age categories (i.e., 6 or 12-h periods) or as a continuous variable would be less artificial and more accurate approach to determine predictive value during the examined postnatal period, that would require such a large sample size that might only be feasible in a future multicenter study. Therefore, prompted by our results, we seek to outline and conduct such a future study in a larger population and on other vendors’ MR scanner, as well as to compare the predictive value for 2-year neurological outcome with that for longer-term (i.e., 7 and 14 years of age) neurodevelopmental outcome, in order to refine and optimize the prognostic power of conventional H-MRS.

## Conclusion

In summary, we propose that conventional brain H-MRS examination has consistently high prognostic value for metabolite ratios NAA/Cr and mI/NAA throughout the first 2 postnatal weeks of newborns with HIE, with the highest predictive power between postnatal days 7–14. Moreover, actual metabolite ratios show the strongest association with the long-term outcome and are not overall significantly affected by gestational age and postnatal age. This implies that, while assessed separately in the outcome groups, gestational age and postnatal age-dependent metabolite variations do appear, and these are strongly outweighed by the consequential outcome, presumably determined by severity of insult.

## Supplementary information

Supplementary information

Supplementary table

Supplementary table
