# Supplementary figures and images for: Predictive performance and metabolite dynamics of proton MR spectroscopy in neonatal hypoxic–ischemic encephalopathy
Source: Pediatr Res. 2021 Sep 6;91(3):581–9. doi: 10.1038/s41390-021-01626-z (PMC8904256; doi:10.1038/s41390-021-01626-z)

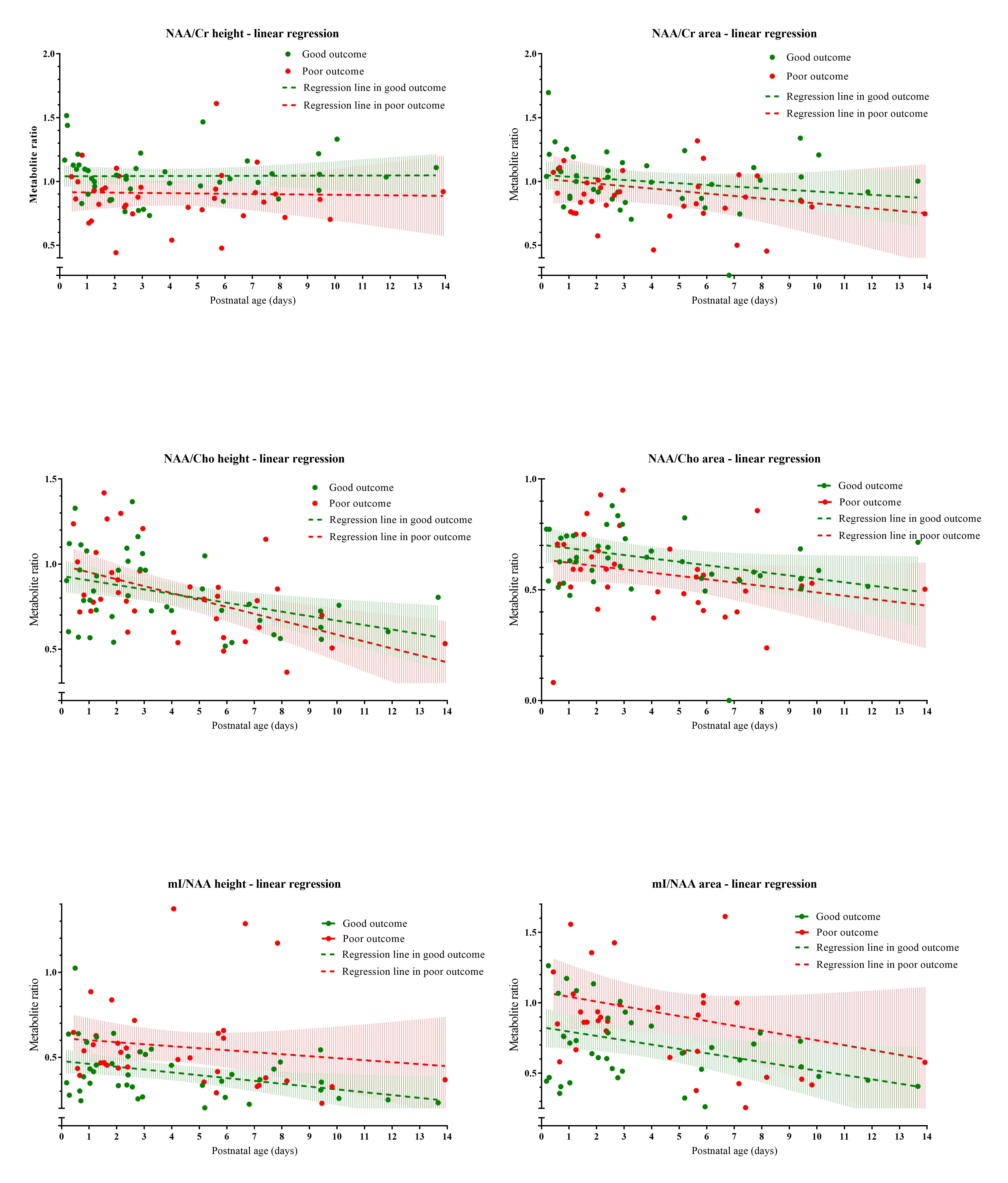

Supplement: Supplementary file 1 — Supplementary information [file 41390_2021_1626_MOESM1_ESM.jpg]
