# Supplementary material for: Predictive performance and metabolite dynamics of proton MR spectroscopy in neonatal hypoxic–ischemic encephalopathy
Source: Pediatr Res. 2021 Sep 6;91(3):581–9. doi: 10.1038/s41390-021-01626-z (PMC8904256; doi:10.1038/s41390-021-01626-z)
Supplement: Supplementary file 2 — Supplementary table [file 41390_2021_1626_MOESM2_ESM.docx]

**Supplementary table. Metabolite ratios detected in HIE newborns having more than one H-MRS examination.**

NAA: N-acetyl-aspartate, Cr: creatine, Cho: choline, mI: myo-inositol.
